# Vectors for multi-color bimolecular fluorescence complementation to investigate protein-protein interactions in living plant cells

**DOI:** 10.1186/1746-4811-4-24

**Published:** 2008-10-15

**Authors:** Lan-Ying Lee, Mei-Jane Fang, Lin-Yun Kuang, Stanton B Gelvin

**Affiliations:** 1 Department of Biological Sciences, Purdue University, West Lafayette, IN 47907-1392, USA; 2 Core Facilities, Academia Sinica, Taipei, Taiwan; 3 Transgenic Plant Core Facility, Academia Sinica, Taipei, Taiwan

## Abstract

**Background:**

The investigation of protein-protein interactions is important for characterizing protein function. Bimolecular fluorescence complementation (BiFC) has recently gained interest as a relatively easy and inexpensive method to visualize protein-protein interactions in living cells. BiFC uses "split YFP" tags on proteins to detect interactions: If the tagged proteins interact, they may bring the two split fluorophore components together such that they can fold and reconstitute fluorescence. The sites of interaction can be monitored using epifluorescence or confocal microscopy. However, "conventional" BiFC can investigate interactions only between two proteins at a time. There are instances when one may wish to offer a particular "bait" protein to several "prey" proteins simultaneously. Preferential interaction of the bait protein with one of the prey proteins, or different sites of interaction between the bait protein and multiple prey proteins, may thus be observed.

**Results:**

We have constructed a series of gene expression vectors, based upon the pSAT series of vectors, to facilitate the practice of multi-color BiFC. The bait protein is tagged with the C-terminal portion of CFP (cCFP), and prey proteins are tagged with the N-terminal portions of either Venus (nVenus) or Cerulean (nCerulean). Interaction of cCFP-tagged proteins with nVenus-tagged proteins generates yellow fluorescence, whereas interaction of cCFP-tagged proteins with nCerulean-tagged proteins generates blue fluorescence. Additional expression of mCherry indicates transfected cells and sub-cellular structures. Using this system, we have determined in both tobacco BY-2 protoplasts and in onion epidermal cells that *Agrobacterium *VirE2 protein interacts with the *Arabidopsis *nuclear transport adapter protein importin α-1 in the cytoplasm, whereas interaction of VirE2 with a different importin α isoform, importin α-4, occurs predominantly in the nucleus.

**Conclusion:**

Multi-color BiFC is a useful technique to determine interactions simultaneously between a given" bait" protein and multiple "prey" proteins in living plant cells. The vectors we have constructed and tested will facilitate the study of protein-protein interactions in many different plant systems.

## Background

Visualization of protein-protein interactions in living cells has become an increasingly important tool for defining protein function and the "web" of proteins constituting the "interactome" [1]. Although *in vivo *protein-protein interactions have been investigated using FRET, BRET, TAP-tagging, and co-immunoprecipitation [2,3], bimolecular fluorescence complementation (BiFC) has more recently added a new technique to the arsenal of measures used to investigate protein-protein interactions. BiFC uses reconstitution of fluorescence from a "split fluorophore" to visualize interaction between two tagged proteins [4,5]. The *Aequorea *green fluorescent protein (GFP) or its wavelength-shifted derivatives can be split in several different places (e.g., between amino acids 154 and 155, or between amino acids 173 and 174). Neither the N-terminal nor C-terminal fragments (either alone or affixed as a translational fusion to other proteins) fluoresces. However, when brought together by interaction of the two affixed proteins, these GFP fragments may fold and reconstitute a fluorescent molecule [4]. Several BiFC systems have recently been described for use in plants [6-8]. Bhat et al. [3] and Ohad et al. [9] have recently reviewed the use of BiFC in plants.

BiFC is conventionally used to visualize the interaction of two proteins. However, there may be instances in which investigators may wish simultaneously to visualize potential interactions between a "bait" protein and a number of "prey" proteins. This can best be accomplished if each of the prey proteins are tagged with different GFP derivative protein fragments that, when reconstituted with the complementary protein fragment, will fluoresce at different wavelengths. Such "multi-color BiFC" reactions were first described by Hu and Kerppola [10] to visualize interactions among domains of different bZIP transcription factors in animal cells.

In this paper, we describe a series of expression vectors to facilitate the use of multi-color BiFC in plant cells. As an example to demonstrate how multi-color BiFC can be used to distinguish different sub-cellular sites of interaction between a bait protein and multiple prey proteins, we have investigated simultaneous interactions between *Agrobacterium *VirE2 protein and two *Arabidopsis *nuclear transport importin α adapter proteins, AtImpa-1 (importin α-1) and AtImpa-4 (importin α-4).

## Results and discussion

### Generation of multi-color BiFC vectors

**Figure 1:**
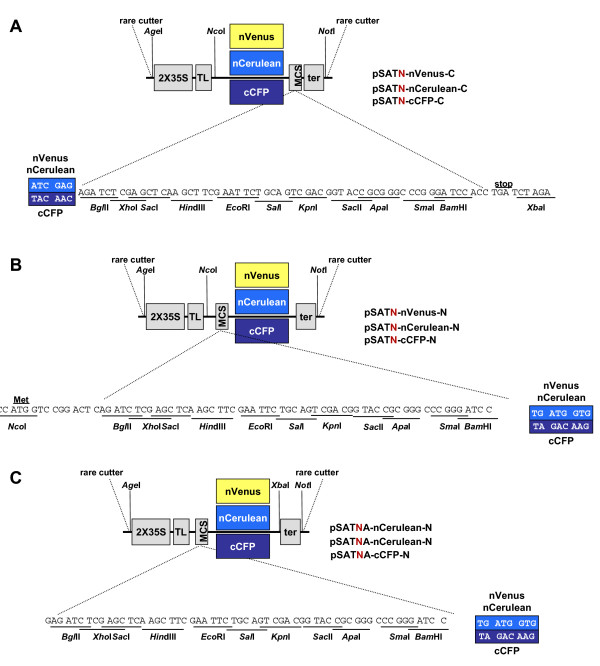
**Schematic diagrams of the multi-color BiFC vectors.** A, Vectors for tagging proteins at their N-termini with autofluorescent protein fragments; B and C, Vectors for tagging proteins at their C-termini with autofluorescent protein fragments. The pSAT-NA series described in Panel C have the upstream *Nco*I site deleted. Note that an ORF fragment tailored to maintain the open reading frame with the autofluorescent protein fragment of the pSAT-C vectors will be out of frame if ligated into pSAT-N vectors.

To facilitate the use of multi-color BiFC in plants, we adapted a previously-described series of pSAT vectors [8]. The pSAT vectors [11] are built in modular fashion, with rare-cutting restriction endonuclease or homing endonuclease sites surrounding an "expression cassette". Each "set" of pSAT vectors is flanked by different rare-cutting sites, and includes a double Cauliflower Mosaic Virus (CaMV) promoter, a Tobacco Etch Virus (TEV) translational leader, a multi-cloning site either preceding or following an autofluorescent protein N- or C-terminal fragment, and a CaMV polyA addition signal. Shyu et al. [12] showed that pairing the C-terminal fragment of cyan fluorescent protein (cCFP) with either the N-terminal fragment of Cerulean (nCerulean) or the N-terminal fragment of Venus (nVenus) results in more intense blue or yellow fluorescence, respectively, than using other GFP-derived autofluorescent protein fragments. "Overlapping" the N- and C-terminal autofluorescent protein fragments additionally increased signal intensity. We therefore used nVenus or nCerulean fragments from amino acids 1–173, and cCFP from amino acids 155–238. Figures 1A–C show the final constructions. Table 1 lists the currently available multi-color BiFC vectors that we have constructed.

**Table 1:** Multi-color BiFC Vectors

**Gelvin lab stock number**	**Plasmid name**	**Autofluorescent protein fragment**	**Rare-cutting site flanking cassette**	**Protein fusion^a^**
E3228	pSAT1-nVenus-C	nVenus	AscI	C-terminal
E3229	pSAT4-nVenus-C	nVenus	I-SceI	C-terminal
E3230	pSAT6-nVenus-C	nVenus	PI-PspI	C-terminal
E3308	pSAT1-nVenus-N	nVenus	AscI	N-terminal
E3231	pSAT1A-nVenus-N	nVenus	AscI	N-terminal
E3310	pSAT4-nVenus-N	nVenus	I-SceI	N-terminal
E3232	pSAT4A-nVenus-N	nVenus	I-SceI	N-terminal
E3233	pSAT6-nVenus-N	nVenus	PI-PspI	N-terminal
E3449	pSAT1-cCFP-N	cCFP	AscI	N-terminal
E3450	pSAT1A-cCFP-N	cCFP	AscI	N-terminal
E3451	pSAT4-cCFP-N	cCFP	I-SceI	N-terminal
E3347	pSAT4A-cCFP-N	cCFP	I-SceI	N-terminal
E3497	pSAT6-cCFP-N	cCFP	PI-PspI	N-terminal
E3307	pSAT1-nCerulean-N	nCerulean	AscI	N-terminal
E3246	pSAT1A-nCerulean-N	nCerulean	AscI	N-terminal
E3309	pSAT4-nCerulean-N	nCerulean	I-SceI	N-terminal
E3247	pSAT4A-nCerulean-N	nCerulean	I-SceI	N-terminal
E3248	pSAT6-nCerulean-N	nCerulean	PI-PspI	N-terminal
E3242	pSAT1-cCFP-C	cCFP	AscI	C-terminal
E3243	pSAT4-cCFP-C	cCFP	I-SceI	C-terminal
E3244	pSAT6-cCFP-C	cCFP	PI-PspI	C-terminal
E3415	pSAT1-nCerulean-C	nCerulean	AscI	C-terminal
E3416	pSAT4-nCerulean-C	nCerulean	I-SceI	C-terminal
E3417	pSAT6-nCerulean-C	nCerulean	PI-PspI	C-terminal
E3519	ocs-bar-RCS2-2 (T-DNA binary vector)	N/A	Multiple rare-cutting sites	N/A

^a^N-vectors indicate that the protein of interest in on the N-terminus and the autofluorescent protein fragment is on the C-terminus; C-vectors indicate that the protein of interest in on the C-terminus and the autofluorescent protein fragment is on the N-terminus.

N/A, not applicable

**Figure 2:**
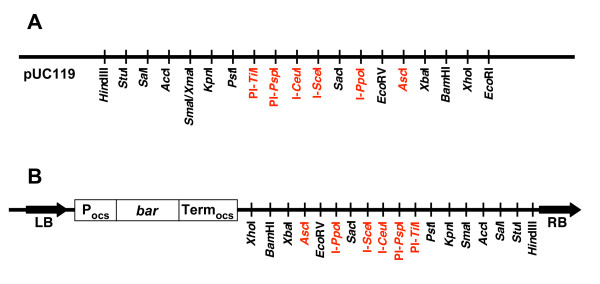
**Schematic diagrams of the multi-cloning sites of the "recipient" plasmids.** A, Plasmid based upon pUC119; B, T-DNA binary vector ocs-bar-RCS2-2 (pE3519). P_ocs_, octopine synthase promoter; *bar*, phosphinothricin/bialaphos/Basta resistance gene; Term_ocs_, octopine synthase polyA addition signal; LB and RB, T-DNA left and right border repeat sequences, respectively.

One of the versatile features of the pSAT series of vectors is that, by using expression cassettes flanked by different rare-cutting enzyme sites, multiple cassettes can be "loaded" into a common replicating plasmid or a T-DNA binary vector for simultaneous introduction into plant cells. Thus, we constructed each new expression cassette in the pSAT1 or pSAT1A vector series (flanked by *Asc*I sites), pSAT4 or pSAT4A vector series (flanked by I-*Sce*I sites), and pSAT6 (flanked by PI-*Psp*I sites). As "recipient" vectors for these expression cassettes, we introduced a multiple rare-cutting site (RCS) sequence into pBluescript KS^+ ^(pBS-RCS), pUC119 (pUC-RCS), and an altered version of the T-DNA binary vector pPZP-RCS2 [13]. pPZP-RCS2 was modified by placing a P_ocs_-*bar*-Term_ocs _selection marker cassette into the *Eco*RI site of the binary vector, near the T-DNA left border, generating ocs-bar-RCS2-2 (pE3519). Figures 2A and 2B show maps of the pUC119 and T-DNA binary vectors, respectively.

### Testing the multi-color BiFC system in tobacco BY-2 protoplasts and onion cells

As proof of concept, we investigated the interaction of *Agrobacterium tumefaciens *VirE2 protein with two members of the *Arabidopsis *nuclear import apparatus, AtImpa-1 and AtImpa-4. VirE2 is a single-stranded DNA binding protein that is exported from *A. tumefaciens *to the plant cell, where it presumably binds to the single-stranded T-DNA (the T-strand). Binding serves to protect the T-strand from nucleolytic degradation within the plant [14,15] and may help direct the T-strand to the nucleus [16,17]. Our laboratory has recently shown that VirE2 can individually interact with the *Arabidopsis *importin α proteins AtImpa-1 and AtImpa-4 in yeast, in tobacco BY-2 cells, and *in vitro*. Individually, VirE2 interacts with AtImpa-1 in the cytoplasm, whereas VirE2 interacts with AtImpa-4 in the nucleus [18]. We therefore conducted multi-color BiFC to determine whether, using this technique, the sub-cellular site of VirE2 interaction with these two importin α isoforms yielded the same results as we had previously determined using individual AtImpa isoforms.

We tagged VirE2 on its C-terminus with cCFP (VirE2-cCFP) in the pSAT1-derived plasmid. Similarly, we tagged AtImpa-1 and AtImpa-4 at their C-termini. Proteins tagged with nVenus were in pSAT4-derived vectors, whereas proteins tagged with nCerulean were in pSAT6-derived vectors. Additionally, we constructed a full-length mCherry expression vector in pRTL2 [19], and an expression vector containing mCherry-VirD2NLS in pSAT6. Both of these mCherry genes were placed under the control of a double CaMV 35S promoter. mCherry localizes to both the nucleus and to the cytoplasm of cells, whereas mCherry-VirD2NLS localizes predominantly to the nucleus [8]. Various combinations of expression cassettes, encoding VirE2, AtImpa-1, and AtImpa-4, were inserted into the corresponding sites of pBluescript-RCS and introduced into tobacco BY-2 suspension cells by electroporation or direct DNA uptake, and into onion cells by particle bombardment. In addition, we co-transfected either a mCherry or a mCherry-VirD2NLS expression cassette to indicate which cells were transfected, and to distinguish various sub-cellular compartments. Experiments in which the VirE2 and AtImpa constructions were co-transfected in the absence of the mCherry markers indicated that expression of mCherry in cells did not alter the sub-cellular sites of localization of the BiFC interaction (data not shown).

**Figure 3:**
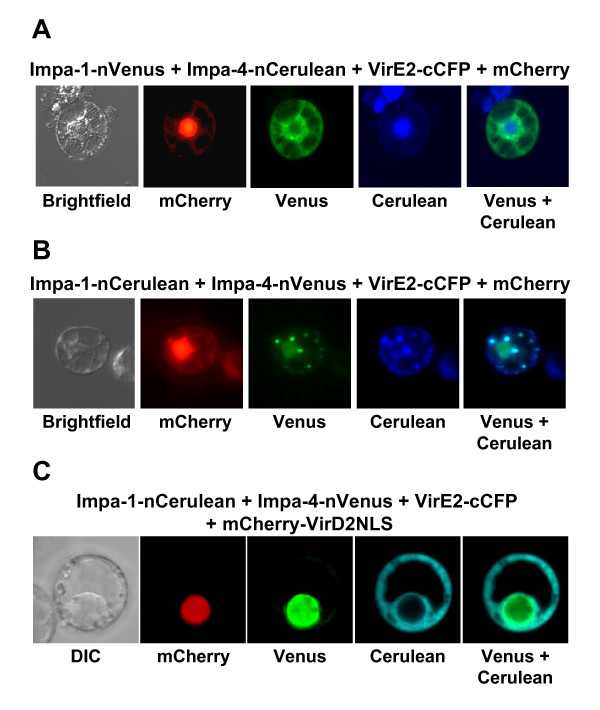
**Multi-color BiFC experiments using tobacco BY-2 suspension culture cells.** In A and B, BY-2 protoplasts were transfected using electroporation and visualized using epifluorescence microscopy. In C, protoplasts were transfected by direct DNA uptake and visualized using laser scanning confocal microscopy. Labels above each set of panels indicate the various constructions introduced into the cells. Labels below each set of panels indicates the filter set/channel imaged. mCherry labels the entire cell, whereas mCherry-VirD2NLS labels only the nucleus. Note that, regardless of the tag, Impa-1 localizes to the cytoplasm and Impa-4 localizes predominantly to the nucleus, with some cytoplasmic staining. In Panel A, the blue signal outside the main imaged cell indicates autofluorescence from dead cells. DIC, differential interference contrast image.

Figure 3 shows results of the tobacco BY-2 transfection assays, as visualized both by epifluorescence and confocal microscopy. AtImpa-1-nVenus interacted with VirE2-cCFP in the cytoplasm, whereas AtImpa-4 simultaneously interacted with VirE2-nCerulean. This latter interaction occurred predominantly in the nucleus, but also weakly in the cytoplasm (see Figure 3A, fourth panel and Figures 3B and 3C, third panels). Nuclear localization of AtImpa-4 was confirmed by co-localization of the nuclear marker mCherry-VirD2NLS.

To assure that the sub-cellular sites of interaction truly reflected properties of the test proteins and not those of the autofluorescent protein tags, we "switched" the tags: AtImpa-1 was now tagged with nCerulean, and AtImpa-4 with nVenus. AtImpa-1-nCerulean continued to interact with VirE2-cCFP in the cytoplasm, whereas AtImpa-4-nVenus interacted with VirE2-cCFP predominantly in the nucleus. Thus, the sub-cellular site of interaction was a function of the test proteins and not of the autofluorescent tag.

**Figure 4:**
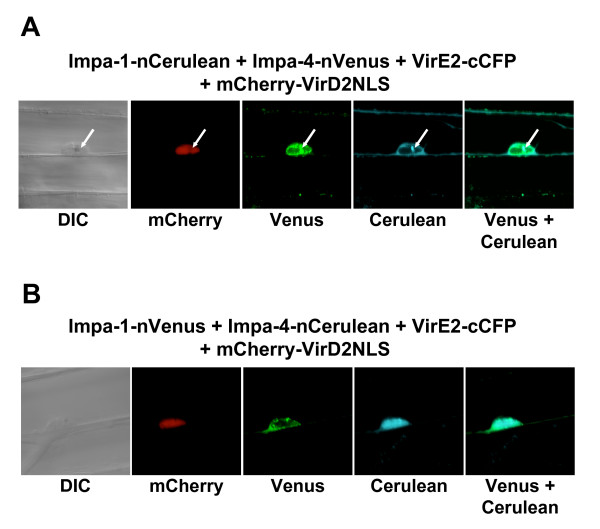
**Multi-color BiFC experiments using onion epidermal cells.** Onion cells were transfected by particle bombardment and visualized using laser scanning confocal microscopy. Labels above each set of panels indicate the various constructions introduced into the cells. Labels below each set of panels indicates the filter set/channel imaged. mCherry-VirD2NLS labels the nucleus. Note that, regardless of the tag, Impa-1 localizes to the cytoplasm and Impa-4 localizes predominantly to the nucleus, with some cytoplasmic staining. In Panel A, the arrows indicate a gold particle in the nucleus. Imaging of gold particles results from 458 nm and 488 nm laser reflection. Because Cerulean images weakly using the Zeiss LSM510 Meta confocal microscope, the Cerulean images in Panels A and B were digitally enhanced by adjusting the brightness and contrast, in accordance with [22]. DIC, differential interference contrast image.

Although *A. tumefaciens *can infect a wide variety of plants, including monocots [20], dicotyledonous plants such as tobacco are more "natural" hosts (i.e., plants which can develop Crown Gall disease). We therefore were interested in determining whether *Agrobacterium *VirE2 protein could interact with *Arabidopsis *importin α proteins in onion, which is not a natural *Agrobacterium *host. We introduced the various combinations of tagged protein-coding genes into onion epidermal cells by particle bombardment and visualized the sub-cellular sites of interaction using laser scanning confocal microscopy. Figure 4 shows that, as in tobacco BY-2 cells, VirE2 interacted with AtImpa-1 in the onion cytoplasm, and with AtImpa-4 predominantly in the onion nucleus. These experiments indicate that the sub-cellular sites of interaction of these proteins are not altered by the particular host, nor by the method of transgene delivery into these hosts.

**Figure 5:**
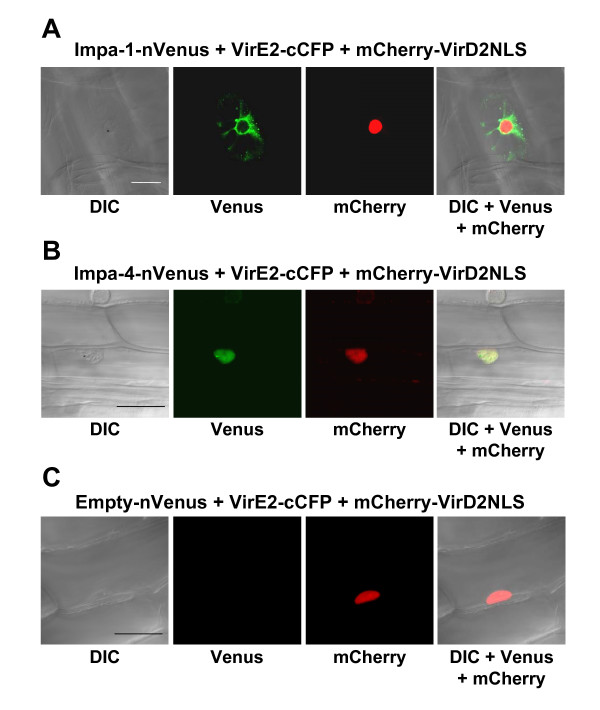
**Control experiments show specificity of multi-color BiFC experiments.** Onion cells were transfected by particle bombardment and visualized using laser scanning confocal microscopy. Labels above each set of panels indicate the various constructions introduced into the cells. Labels below each set of panels indicates the filter set/channel imaged. mCherry-VirD2NLS labels the nucleus. In Panels A and B, respectively, VirE2-cCFP interacts with Impa-1-nVenus or Impa-4-nVenus expressed individually. Localization of yellow fluorescence is identical to that seen when both prey proteins are co-expressed with VirE2-cCFP. Note that in Panel C, yellow fluorescence is not reconstituted in the absence of interacting bait and prey proteins. DIC, differential interference contrast image. Size bars indicate 50 microns.

To assure that, using these vectors, interaction of a bait protein in the presence of multiple prey proteins does not differ from interaction of a bait protein in the presence of a single prey protein, we co-expressed VirE2-cCFP with either AtImpa-1 or AtImpa4. Figures 5A and 5B show that, as described above, VirE2 interacts with AtImpa-1 in the cytoplasm and with AtImpa-4 predominantly in the nucleus of bombarded onion cells. In addition, we tested whether fluorescence complementation could occur in the absence of interacting bait and prey proteins. Figure 5C shows that when VirE2-cCFP was co-expressed with nVenus (empty-nVenus), no yellow fluorescence was observed. To assure that the onion cells had received the various BiFC constructs, we included in this latter control experiment a mCherry-VirD2NLS expression construct cloned into the same vector as the BiFC expression cassettes. Red fluorescence was detected and localized to the nucleus, indicating that the onion cell had received the various BiFC constructs. Thus, we did not detect reconstituted yellow fluorescence in the absence of appropriately tagged bait and prey proteins.

## Conclusion

We have designed and tested a set of vectors that will be a useful tool for scientists to investigate protein-protein interactions in living plant cells. Proteins of interest can readily be tagged with a number of autofluorescent protein fragments in gene expression cassettes. These cassettes can simultaneously be introduced into plants as separate plasmids, or "loaded" in different combinations into a common plasmid for introduction into plant cells using either naked DNA- or *Agrobacterium*-mediated transformation. Along with various forms of mCherry to mark sub-cellular compartments, these multi-color BiFC vectors will be useful tools for investigating interactions among multiple protein partners. We recognize that over-expression of proteins using strong promoters to drive expression of the encoded genes can affect the extent and nature of interactions with other proteins. However, we have shown that the nuclear localization of the AtImpa-4/VirE2 protein pair using strong promoters is identical to that seen when using the native AtImpa-4 promoter [18].

The expression vectors and recipient plasmids described in this study can be obtained by contacting SBG gelvin@bilbo.bio.purdue.edu. DNA sequences and maps for the various vectors can be found at: .

## Methods

### Vector construction

PCR amplified nVenus, nCerulean, and cCFP fragments were initially cloned into pBluescript II KS^+^. After sequences were confirmed, all three fragments were released from the vector by digestion with the appropriate restriction endonucleases and cloned into pSATN(A) vectors. Primers used for nVenus and nCerulean were: FP-1C (*Nco*I): TTA ACC ATG GTG AGC AAG GGC GAG; FP-2C (*Bgl*II): AGA TCT CTC GAT GTT GTG GCG GAT; FP-3N (*Bam*HI): TAT GGG ATC CTG ATG GTG AGC AAG GGC GAG; FP-4N (*Xba*I): GCG GGA TCT AGA CTA CTC GAT GTT GTG GCG. Primers used for cCFP were: cCFP-1 (*Nco*I): AAT ACC ATG GAC AAG CAG AAG AAC GGC; cCFP-2 (*Bgl*II): ATT GGC AGA TCT CTT GTA CAG CTC GTC CAT; cCFP-3 (*Bam*HI): ACA GAA TGG ATC CTA GAC AAG CAG AAG AAC GGC; cCFP-4 (*Xba*I): A CCT TCT AGA TCA CTT GTA CAG CTC G. Forward primers are FP-1C, FP-3N, cCFP-1, and cCFP-3. Reverse primers are FP-2C, FP-4N, cCFP-2, and cCFP-4. To perform PCR, we paired the *Nco*I forward primer with the *Bgl*II reverse primer (Figure 1A), and the *Bam*HI forward primer with the *Xba*I reverse primer (Figures 1B and 1C).

### Plant transformation

For electroporation experiments, protoplasts were isolated from five-day old *Nicotiana tabacum *BY-2 suspension cells. Suspension cultures were grown at 23°C with shaking (130 rpm) in a medium containing Murashige and Skoog salts [21] supplemented with 1 mg/L thiamine-HCl, 370 mg/L KH_2_PO_4_, 30 g/L sucrose, and 2 mg/L2, 4-dichlorophenoxyacetic acid, pH 5.7. Cells were sub-cultured once per week by adding 2.5–3 ml of inoculum to 50 ml of fresh medium in 250 ml Erlenmeyer flasks. The 50 ml of suspension culture was centrifuged at 250 × g at room temperature for 5 min in a Sorvall GLC-2 centrifuge, and the pellet (15 ml packed cell volume) was re-suspended in 50 ml of protoplast isolation solution containing 7.4 g/L CaCl_2 _·2H_2_O, 1 g/L NaOAc, and 45 g/L mannitol supplemented with 1.2% cellulose R10 (Onazuka) and 0.6% Macerozyme (Duchefa), pH 5.7. Approximately 15 ml of suspension culture was transferred into three 20 × 100 mm sterile Petri dishes and incubated in the dark with a gentle shaking (40 rpm) at room temperature for 4 hours. The protoplasts were washed twice with protoplast isolation solution and the pellet was re-suspended in 50 ml of floating solution (99 mg/L myo-inositol, 2.88 g/L L-proline, 100 mg/L enzymatic casein hydrolysate, 102.6 g/L sucrose, 97.6 mg/L MES buffer, 4.3 g/L MS salts, 1 mg/L thiamine-HCl, 370 mg/L KH_2_PO_4_, pH 5.7). Protoplasts (floating on the top of the solution) were transferred to a new tube, washed twice, and suspended in 50 ml of electroporation solution (10 mM NaCl, 4 mM CaCl_2_·2H_2_O, 120 mM KCl, 10 mM HEPES, 0.6 M mannitol, pH 7.2). Cells were incubated at 42°C for 5 min and kept in ice for 10 min before electroporation. Aliquots of protoplasts containing approximately 3 × 10^6 ^cells/ml were used for electroporation. ~20 μg of each plasmid DNA were added to 300 μl of protoplasts in a tube and placed on ice. The electroporation was conducted using a BioRad Gene Pulser apparatus at 0.16 kV, with the Pulse Controller set to infinity and the capacitance extender set to 960 μFD. After 10 min incubation on ice, the protoplasts were transferred into 10 ml of BY-2 culture medium supplemented with 0.4 M mannitol and incubated overnight.

For direct DNA uptake experiments, 20 ml BY-2 suspension cells were transferred to a sterile conical centrifuge tube and centrifuged at 3000 rpm for 10 min. The pelleted cells were suspended in 10–20 ml protoplast digestion enzyme solution (1.2% Cellulase Onozuka RS [Duchefa] and 0.6% Macerozyme R-10 [Duchefa] in 10 mM CaCl_2_·2H_2_O, 12 mM NaOAc, 11% mannitol, pH 5.7) and incubated in the dark with shaking (40 rpm) for 3–4 hr at room temperature. The protoplasts were filtered through 40 μm nylon mesh and centrifuged in a 50 ml conical tube at 250 × g for 5 min. The protoplasts were collected and suspended in 10 ml protoplast floating solution (per liter: 99 mg myo-inositol, 2.88 g L-proline, 100 mg enzymatic casein hydrolysate, 102.6 g sucrose, 97.6 mg MES buffer, 4.3 g MS salts, 1 mg Vitamin B1, 370 mg KH_2_PO_4_, pH 5.7) and centrifuged at 250 × g for 10 min. Protoplasts floating in this solution were removed and 10 ml W5 solution (154 mM NaCl, 125 mM CaCl_2_, 5 mM KCl, 2 mM MES, pH 5.7) was added. The solution was centrifuged at 250 × g for 5 min, and the protoplasts pelleted. Protoplast concentration was adjusted to 1 × 10^6^/ml and the solution incubated on ice for 30 min. The protoplasts were again centrifuged at 250 × g for 5 min and suspended at a density of 1 × 10^6 ^cells/ml in MMg solution (0.6 M mannitol, 15 mM MgCl_2_, 4 mM MES, pH 5.7). DNA (10 μg in 10 μl) was added to 100 μl protoplasts, followed by addition of 110 μl PEG solution (per ml: 0.4 g PEG 4000 [Fluca], 0.6 ml 1 M mannitol, 100 μl 1 M CaCl_2_), and the protoplasts incubated at room temperature for 30 min. After addition of 1 ml W5 solution, the protoplast suspension was centrifuged at 250 × g for 5 min. The protoplast pellet was suspended in 1 ml incubation solution (per liter: MS salts and vitamins, 2 mg/L 2,4-D, 3% sucrose, 0.4 ~ 0.6 M mannitol) and incubated in the dark for 16 hr at 26°C.

Particle bombardment of onion epidermal peel layers was carried out using a Biolistic Particle Delivery System (Bio-Rad) PDS-1000. Samples (whole onion from which the dry outer layer was removed) were sterilized in ~300 ml 2% NaOCl and 2–3 drops Tween-20 for 15 min. The tissue was washed with sterilize H_2_O at least five times. The upper epidermal layer of the onion was removed, cut into 2 × 2 cm squares, and placed on a plate containing 1/2 MS medium. 5 μg of each plasmid DNA was used in all experiments. Gold particles size was 1.6 μm (INBIO GOLD). 0.15–0.2 mg particles/per shot were used with a chamber vacuum of 27 in Hg. Particles were accelerated with a pressure of 1100 psi. The distance between the projectile source and the samples was 6 cm.

### Microscopy

Transfected cells were imaged using a Nikon Eclipse E600 fluorescence microscope, or a Zeiss LSM510 Meta confocal microscope. For confocal microscopy, the objective lens was a C-Apochromat 63×/1.2 W corr. Channel specifications were as follows:

Multi Track: Channel 1 (Venus track): Argon laser; Excitation: Line active 488 nm; Transmission 8%; Main Beam Splitter 1: 488/543/633; Beam Splitter 2: 545; BP 500–530IR; Detector Gain: 620; Amplifier Offset: -0.1.

Multi Track: Channel 2 (mCherry track): HeNe laser; Excitation: Line active 543 nm; Transmission 100%; Main Beam Splitter 1: 488/543/633; Beam Splitter 2: 545; BP 565–615IR; Detector Gain: 550; Amplifier Offset: -0.1.

Multi Track: Channel 3 (Cerulean track): Argon laser; Excitation: Line active 458 nm; Transmission 20%; Main Beam Splitter 1: 458; Beam Splitter 2: mirror; BP 480–520IR; Detector Gain: 550; Amplifier Offset: -0.1.

DIC channel: HeNe laser 543 nm; Detector Gain: 198; Amplifier Offset: -0.1

## Competing interests

The authors declare that they have no competing interests.

## Authors' contributions

LYL designed and constructed the vectors, tested the vectors in electroporated tobacco BY-2 protoplasts, and analyzed the data. MJF conducted confocal microscopic studies on transfected tobacco BY-2 and onion epidermal cells. LYK conducted plant cell transfections on tobacco BY-2 protoplasts by naked DNA uptake, and on onion epidermal cells by particle bombardment. SBG helped design the experiments, analyzed the data, and wrote the manuscript. All authors read and approved the final manuscript.
